# Role of CXCL13 in the formation of the meningeal tertiary lymphoid organ in multiple sclerosis

**DOI:** 10.12688/f1000research.14556.3

**Published:** 2018-10-04

**Authors:** Ana C. Londoño, Carlos A. Mora

**Affiliations:** 1 Instituto Neurológico de Colombia-INDEC, Medellín, Colombia; 2 Department of Neurology, MedStar Georgetown University Hospital, Washington, DC, 20007, USA

**Keywords:** multiple sclerosis, chemokines, CXCL13, B cells, tertiary lymphoid organ, meninges

## Abstract

Immunomodulatory therapies available for the treatment of patients with multiple sclerosis (MS) accomplish control and neutralization of peripheral immune cells involved in the activity of the disease cascade but their spectrum of action in the intrathecal space and brain tissue is limited, taking into consideration the persistence of oligoclonal bands and the variation of clones of lymphoid cells throughout the disease span. In animal models of experimental autoimmune encephalomyelitis (EAE), the presence of CXCL13 has been associated with disease activity and the blockade of this chemokine could work as a potential complementary therapeutic strategy in patients with MS in order to postpone disease progression. The development of therapeutic alternatives with ability to modify the intrathecal inflammatory activity of the meningeal tertiary lymphoid organ to ameliorate neurodegeneration is mandatory.

## Introduction

**Figure 1.**
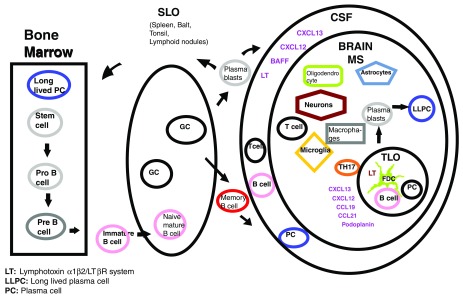
B cells lineage from bone marrow to CNS. B-cells originating in the bone marrow exit toward the blood stream as immature B-cells; they enter the SLO and specialize in the germinal centers producing memory B cells and plasmablasts, which in pathologic conditions, are able to gain access to the CNS. The TLO is formed in the meninges during chronic inflammation in the deep brain cortical sulci and share organogenesis with the SLO ^24^. Podoplanin and the Th17 signature cytokine IL-17 have been associated with ectopic lymphoneogenesis in human diseases whereas BAFF is a key factor for mutation and survival of B cells which is produced by astrocytes in the CNS ^3, 25^. **BAFF:** B-cell activating factor of the tumor necrosis factor family; **Balt:** bronchial associated lymphoid tissue; **CCL19:** chemokine (c-c motif) ligand 19; **CSF:** cerebrospinal fluid; **CXCL13:** chemokine (C-X-C motif) ligand 13; **FDC:** follicular dendritic cells; **GC:** germinal center; **LT:** lymphotoxin α1β2/LTβR system; **LLPC:** long lived plasma cells; **MS:** multiple sclerosis; **PC:** plasma cell; **SLO:** secondary lymphoid organ; **TLO:** tertiary lymphoid organ.

Although disease modifying therapy (DMT) agents in multiple sclerosis (MS) have contributed to reduction of neuroinflammation, they have not succeeded in the prevention of progression of disease. Inflammation is the appropriate tissue response to infection, autoimmunity, cancer, injury and allograft transplantation ^1^. When inflammation does not resolve appropriately, a prolonged immune response persists leading to tissue destruction and loss of function ^1^. Chronic infiltration by immune cells in the meninges is believed to form transitory lymphoid cell aggregates which simulate secondary lymphoid organs (SLO), and are known as meningeal tertiary lymphoid organs (mTLO) which play an important role in the pathogenesis of autoimmunity ^1, 2^. The mTLO seem to play a role in the intrathecal activity of immune system cells in MS ^3^. The SLO, such as lymph nodes, show a cellular organization that includes germinal centers (GC) containing antibody secreting and proliferating B-cells with follicular dendritic cells (FDC), a T-cell zone that incorporates naïve cells from the blood stream, high endothelial venules for extravasation of lymphocytes, and a stromal cell network that provides chemokines and extracellular matrix for cell migration and structural integrity ^1^. Chemokines are a family of proteins with the specific property of regulating leukocytes in the immune system and they may play a role in neurotransmission and neuromodulation ^4^. Leukocyte trafficking is mediated by inflammatory chemokines in inflamed tissues and by homeostatic chemokines in lymphoid sites ^5^ ( Figure 1). In this review, we focus on the role that CXCL13 (also known as B cell attracting chemokine [BCA-1], C-X-C motif ligand 13, or B lymphocyte chemoattractant [BLC]) plays in the organization of the mTLO in MS.

## In normal conditions, the SLO acquire information and prepare for immune defense

The SLO have a genetically determined pattern of development and programming that allows trapping and concentration of foreign antigens to initiate an adaptive immune response ^1^. Mucosal associated and non-encapsulated lymphoid tissue (including the Peyer’s patches, adenoid tissue of the nasopharynx, tonsils, and the bronchial associated lymphoid tissue), together with lymphoid nodes and spleen, constitute the SLO ^6, 7^. The lymph node cortex contains clusters or primary follicles that include packaged B cells and FDC, whereas the node para-cortex has a lesser number of dendritic cells (DC) and T cells ^6^. Generation of B cells with ability to produce auto antibodies usually occur in physiological conditions ^8^. These auto antibodies are low affinity IgM, which exhibit a wide spectrum of reactivity and strong preference for soluble self-antigens on the cell surface ^8^. Auto reactive low affinity B cells suffer apoptosis being unlikely they represent danger in normal conditions ^8^.

## Lymphoid cells are able to learn and exchange information at the GC

**Figure 2.**
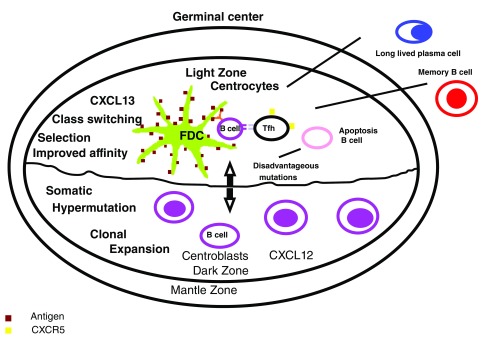
The germinal center. B-cells enter the dark zone of the germinal center (centroblasts), a step which depends on the expression of CXCR4 in the surface, where the cells go through proliferation and somatic hypermutation (SHM). Subsequently, the cells migrate to the light zone (centrocytes) where they capture antigens through the mutated B cell receptors and are internalized for presentation to the T cells. The centrocytes differentiate from the centroblasts by the level of expression of surface proteins. Centrocytes are CXCR4 ^low^, CD83 ^high^, CD86 ^high^ and the centroblasts are CXCR4 ^high^, CD83 ^low^ y CD86 ^low^. The fluctuation between centroblasts and centrocytes is part of a synchronized cellular program which permits a temporal separation of the processes of mitosis and SHM from selection. The functional output of the TLO, in comparison to the SLO, could result from the dysregulated nature of their GC response supporting a breakout of autoimmune variants and the development of long lasting humoral autoimmunity characterized by presence of B cells with minimal memory and LLPC ^15, 16^. **FDC:** follicular dendritic cells; **LLPC:** long lasting plasma cells; **Tfh:** T follicular helper cells.

The GC present remarkable lymphocytic mitosis within SLO follicles ^9^. Weyand *et al*. stated the GC are critical in the development of the B-cell normal immune response by driving-in cell division and maturation, B-cell selection with high affinity for immunoglobulin receptors and differentiation of B-cells and plasma cells (PC) ^2^. Real time imaging technology has allowed visualization of the transit of the B cells from the dark zone to the light zone, and vice versa, during the maturation of the GC ^9– 11^. The GC light zone displays a predominance of FDC and follicular T-helper (Tfh) cells, whereas the dark zone contains closely packed lymphocytes and stromal cells ^9, 12^. The chemokine receptor CXCR4 is required for the positioning of the B cells in the dark zone where its ligand, CXCL12, is more abundant and is produced by stromal cells ^13^. At the light zone, CXCL13 chemokine is concentrated in the FDC processes and, in conjunction with CXCR5, they contribute to the accumulation of B cells in this zone ^12, 13^. T-cells in the GC are essential to maintain signaling and represent approximately 5–20% of cell population ^9^. Tfh cells are characterized by the expression of CXCR5 and ICOS, which has an inducible T cell co-stimulatory effect ^9, 14^. B cell growth and differentiation at the germinal center are supported by IL10 and ICOS ^14^. Within the light zone, the three possible different outcomes for the centrocytes include death due to apoptosis; differentiation into memory B-cell or long lived plasma cells (LLPC); and re-entrance to the dark zone for a further round of cell mutation and selection ^15^. The relevant function of the GC is, most likely, the primary production of memory B-cells and LLPC ^15, 16^ ( Figure 2). Recent studies analyzing IgG heavy chain variable region genes in B cells from MS patients revealed that B cells are able to enter and exit the blood brain barrier (BBB) in order get exposed to somatic hypermutation (SMH) at the GC ^17– 21^.

## Chemokines direct traffic of lymphocytes during the cell search for specific information

The induction of lymphoid chemokines, depends on lymphotoxin β (LT-β) and the tumor necrosis factor α (TNF-α) signaling on stromal cells and FDC ^22^. Lymphotoxin α1β2 (LTα1β2) is expressed in the surface of B and T cells in the adult immune system and ligates to the lymphotoxin β receptor (LTβR) in reticular stromal cells thus inducing expression of lymphoid chemokines, such as CCL19, CCL21 and CXCL13 ^23^. These chemokines regulate the homeostatic traffic of lymphocytes in lymphoid organs and their distribution in the GC ^23^. Homeostatic chemokines promote secretion of LTα1β2 by T and B cells, establishing a feedback loop that perpetuates the recruitment of lymphocytes and positional organization in the GC ^1^. The chemokine CXCL13 has the following relevant properties:

**1.**  CXCL13 increases its own production by stimulating the growth of FDC after regulating LTα1β2 on the membrane of B cells ^5, 26^.
**2.**  CXCL13 is produced in the SLO by FDC and macrophages and is an important chemoattractant to the CNS ^27, 28^.
**3.**  Follicular stromal cells express CXCL13, which is needed for nesting CXCR5 ^+^B cells and a subset of T cells in the follicular compartment ^6^.
**4.**  CXCL13 primarily works through CXCR5 expressed in mature B lymphocytes ^8^, CD4+ Tfh ^29^, CD4+ Th17 cells ^30^, minor subset of CD8+ T cells and activated tonsil Treg cells ^8, 30^.
**5.**  CXCL13 has no relation with CD138+ and CD38+ plasmablasts, and PC ^17^.

Stromal cells from the T cell zone express the chemokines CCL19 and CCL21, which share the receptor CCR7 that directs naïve, central memory T cells and DC to the T cell compartment ^6, 31^. CXCR5 is expressed in 20 to 30% of CD4+T cells in blood and CSF, and virtually in all B cells in blood and the majority of B cells in the CSF compartment ^32^. Mice lacking CXCL13, or its receptor CXCR5, fail to develop peripheral lymph nodes ^1^. Khademi *et al*. determined the concentration of CXCL13 in CSF of individuals with MS, other neurological diseases including viral and bacterial infection, and healthy controls finding higher levels of the chemokine in subjects with infections followed to a lesser extent by the patients with MS ^33^. The levels of CXCL13 correlated negatively with disease span, concluding that early determination of CXCL13 might predict prognosis of disease ^33^.

## Could the TLO become an operation center with ability to magnify an autoimmune response?

By maintaining antibody diversity, B cell differentiation, isotype switching, oligoclonal expansion, and local production of autoreactive PCs, the TLO perpetuate disease in response to environmental inputs ^34^. Lymphoid organogenesis and formation of mTLO may be facilitated by expression of lymphotoxin α (LT-α) at the external layer of meningeal inflamed vessels leading to the compartmentalization of the immune response in MS ^17, 35^. It has been postulated that the perpetuation of neuroinflammation and disease progression results from mTLO induced differentiation and maturation of antigen specific effector lymphocytes ^28^. The identification of independent centroblasts in the CSF of MS patients has suggested that there is an intrathecal B cell differentiation which is not dependent on the immune activity in the blood compartment ^17^. The TLO, besides SLO, provide a thriving environment where PC differentiate from plasmablasts ^6, 28^. In the absence of recirculating immune cells from the periphery, the TLO exerts its remarkable ability to remain active for several weeks ^36^. Therefore, the neutralization of TLO could play a significant role by blocking the re-emergency of auto reactive clones that could be able to drive relapses or resistance to therapy ^36^. Th17 cells, Tfh and a subtype of activated B cells, which are critical in systemic inflammation related with presence of TLO, are strongly associated with MS progression ^37^. In an animal model of experimental autoimmune encephalitis (EAE), Peters *et al.* found the following: 1) Expression of CXCL13 in the CNS of Th17 cells recipient mice; 2) evidence that IL17 and the surface molecule podoplanin contribute to the development of ectopic lymphoid follicle in target organs; and 3) evidence of GC-like reactions in some of the mTLO as suggested by the presence of CXCL13, PNA- and GL7- positive T and B cells and plasma cells ^25^. By using next-generation RepSeq analysis, Lehmann-Horn *et al*. demonstrated the presence of SHM and class switch recombination (CSR) in the mTLO B-cell aggregates which had been initiated by activation of the induced cytidine deaminase enzyme (AICDA) in the Thx2D2 mice animal model of chronic EAE ^38^. The B-cell repertoires found at the mTLO were different than those found at the SLO suggesting that the mTLO represent autonomous sites of immune activity and that GC activity in the mTLO was antigen driven ^38^.

## In absence of CXCL13, a reduced inflammatory response emerges from studies on animal models and human pathology

Disorganized B cell follicles in SLO have shown reduced capacity to originate natural antibody responses in CXCL13-/- mice ^26, 39^. Deficiency of CXCL13 results in a moderate course of disease characterized by a better recovery with attenuation of white matter inflammation and gliosis during the acute and chronic stage of EAE ^40^. Krumbholz *et al*. showed there was a direct correlation between CXCL13 levels and the number of B cells, T cells and plasmablasts in the CSF of MS patients ^5^. Clonal expansion and SMH of B cells have been observed in the CSF of patients with MS ^41^. CXCL13 was upregulated in active MS lesions but not in chronic inactive lesions and, in a similar range, in the serum of patients with relapsing remitting MS (RRMS) and control subjects indicating the intrathecal production of this chemokine ^5^. CXCL13 was identified by immunohistochemistry in intrameningeal B-cell follicles, but not in the cerebral parenchyma, of chronic active or inactive MS lesions ^42^. Patients with clinically isolated syndrome, who had shown conversion to clinically definitive MS within 2 years, had high levels of CXCL13 in the CSF ^33, 43, 44^. Elevated levels of CXCL13 in CSF have also been reported in patients with RRMS compared to controls and the CSF levels have been significantly increased during relapses but declining after initiation of B cell depleting therapy ^22, 33, 45^.

## Facts learned from the role of CXCL13 in other autoimmune diseases

Chronic and active inflammation in target organs such as thymus, thyroid, synovial tissue and salivary gland can be driven by formation of TLO in the corresponding target organs ^2^. Expression of CXCL13 in pancreatic tissue has been associated with formation of ectopic lymphoid follicles and the induction of cascade events leading to diabetes in a transgenic mice model ^46^. In patients with Helicobacter pylori the blockade of CXCL13 has prevented the development of mucosa-associated lymphoid tissue (MALT) lymphoma and the propagation of inflammation by CXCL13 have been documented in non-lymphoid tissue ^47^. Although formation of pulmonary lymphoid follicles has been seen in patients with complicated rheumatoid arthritis, idiopathic pulmonary hypertension and Sjogren’s syndrome, the presence of lymphoid follicles has correlated with positive outcome in patients with lung cancer and infections of the respiratory tract ^48^. Lung B cells are a major source of CXCL13 and it has a positive role in the lymphoid neogenesis in chronic obstructive pulmonary disease through a LT receptor and toll-like receptor signaling ^48^.

## Possible cellular sources of CXCL13 in the CNS

CXCL13 plays an important role in the formation of the GC in ectopic lymphoid follicles of several organs affected by inflammatory or autoimmune disease, or by infection ^1^. Important sources of CXCL13 are follicular stromal and FDC ^26, 49, 50^. In human lung tissue of patients with COPD, Litsious *et al.* found that stromal cells and DC produce CXCL13 upon stimulation by lymphocytes –mainly B cells- that express LT thus acquiring lymphoid tissue inducer (LTi) cells function in the TLO ^48^. The LT stimulates the expression of CXCL13 by stromal cells mainly through the LTβ receptor in the SLO ^13, 26, 48, 51^. Pikor *et al*. reported the production of CXCL13 by meningeal stromal cells when they are in the presence of LTβR in the relapsing-remmiting model of EAE in SJL/J mice ^52^. The source of intrathecal production of CXCL13 during neuroinflammation has not been determined with certainty. However, stromal cells within the B cell follicles have been considered to be responsible for the chemokine production and the notion of simple passive transfer from the blood stream to the intrathecal compartment due to dysfunction of the BBB has been detracted ^13, 26, 51, 53^. According to Essen and collaborators, stromal cells in the meninges could produce CXCL13 in special circumstances and drive the focal accumulation and organization of lymphoid cells in specific sites ^51^. In their study, they were able to demonstrate that microglia cells, *in vivo* and *in vitro*, are the main producers of CXCL13 in acute neuroinflammation induced by the Sindbis virus, which is not associated with demyelination ^51^. They also found that type-1 interferon could suppress the production of CXCL13 by microglial cells ^51^. In the rhesus macaque model of neuroborreliosis, Ramesh *et al*. and Narayan *et al*. found that infiltrating microglia and macrophage/DC myeloid cells could be one source of CXCL13 in the CNS during inflammation ^54, 55^. In biopsy specimens from patients with primary CNS lymphoma, Smith and collaborators encountered the following: 1) FDC were not present in the analyzed specimens; 2) there was expression of CXCR5 and CXCL13 in malignant B cells with positive production of BCA-1 (CXCL13) mRNA; and 3) CXCL13 was present in endothelial vascular cells which had a negative production of BCA-1 (CXCL13) mRNA by *in situ* hybridization, a finding that could be attributed to transcytosis ^56^. In different types of EAE, CXCL13 and BAFF mRNA transcripts were found to be significantly upregulated in the CNS of mice which developed the relapsing-remitting and the chronic-relapsing courses of disease opposite to those which developed a chronic progressive course. Besides, cells expressing CXCL13 were exclusively found in the brain stem meninges where infiltrating leukocyte proliferation was intense and vascular endothelial cells did not express CXCL13 ^57^. In specimens from patients with giant cell arteritis, arterial TLO with FDC precursors and lymphoid ducts were detected in the medial layer of the temporal arteries expressing CXCL13, BAFF, APRIL, IL 7, IL 17 and LTβ ^58^.

## A forthcoming research task: How early are the mTLO formed in the lifespan of MS?

Pikor *et al*. conducted studies in the animal model of relapsing-remitting EAE (SJL/J mice) an observed that at the onset of disease the TLO predominantly have T lymphocytes whereas in the acute an relapsing phase, the meningeal aggregates exhibited both T and B cells ^52^. Meningeal infiltrates can be disperse or well organized encompassing mTLO, whose lifespan is unknown ^28, 42^. The presence of follicles containing proliferating B cells, T cells, PC and FDC that express CXCL13 in the proximity of inflamed blood vessels in the meninges of patients with secondary progressive MS (SPMS) has been documented ^42^. The mTLO correlated with neuronal loss, adjacent cortical demyelination and a more rapid progression of disease ^22^. Patients with SPMS with positive mTLO have shown wide gray matter demyelination associated with loss of neurons, oligodendrocytes, and astrocytes; cortical atrophy, and microglial activation in the outer layer of the cortex ^59, 60^. It remains to be determined whether the formation of mTLO depends on the subtype of disease or it is the result of inflammation or consequence of chronicity ^36^.

## Could CXCL13 be neutralized by direct action on itself, its receptor (CXCR5) or the lymphotoxin β (LT-β)?

A novel therapeutic monoclonal antibody against CXCL13 (Mab 5261 and Mab 5261-muIg) has been shown to induce functional *in vitro* inhibition of the chemokine in humans and mice ^8^. An LTβ receptor blocking immunoglobulin inhibits CXCL13 interactions, suppresses the formation of mTLO in the CNS and ameliorates the symptoms of EAE in rodents ^23^. In EAE induced by the transfer of myelin-specific Th17 cells (Th17 EAE), Quinn *et al*. confirmed a role of Tfh cells by blocking Tfh trafficking using antibody against CXCL13 and found that this treatment significantly reduced expression of disease ^61^. Some DMT available for the treatment for MS ameliorate levels of CXCL13, but the mechanisms by which it occurs are not completely understood. In patients with RRMS treated with natalizumab, a significant reduction in CXCL13 in CSF was observed in comparison to β-interferon ^62^. In another study, Novakova *et al*. evaluated the effect of treatment with fingolimod in CSF biomarkers, including CXCL13, of MS patients who had previously been on β-interferon, glatiramer acetate, teriflunomide (and had to switch therapy because of breakthrough disease activity) or natalizumab (who had to switch due to risk of PML) observing significant reduction of CXCL13 in the CSF of patients in both groups ^63^. Also, Alvarez *et al*. found that in patients with active RRMS, in spite of treatment with β-interferon or glatiramer acetate, the administration of rituximab led to a normalization of the CSF level of CXCL13 in the majority of patients, thus suggesting that high levels of CXCL13 in CSF at baseline could predict a forthcoming therapeutic response to B cell depletion ^64^. Piccio *et al*. found that in patients with RRMS treated with IV rituximab, concomitant with either β-interferon or glatiramer acetate, there was a reduction of CXCL13 and CCL19 in CSF, which correlated with significant reduction of B cells (95%) and T cells (50%) in CSF ^32^. Perry *et al*. found intrathecal reduction of CXCL13 (50.4%) and IgG index (13.5%) resulting from inhibition of development of LTi cells in patients with MS treated with daclizumab ^65^. Braendstrup *et al*. reported the case of a patient with MS who had undergone allogenic hematopoietic stem cells transplant for treatment of follicular lymphoma and who after two years presented negative determination of oligoclonal bands and detectable CXCL13 in CSF ^66^. Esen *et al*. suggested that blockade of CXCL13 could be a possible therapeutic target in EAE with advanced state of inflammation ^51^. However, past experience manipulating some of the chemokines in the treatment of MS has been unfavorable ^67, 68^. Atacicept is a recombinant fusion protein that ligands to the cytokines BLys/BAFF and APRIL which are involved in the differentiation, maturation and survival of B cells ^67, 68^. Although atacicept does not accomplish depletion of progenitor or memory B cells ^68^, it has the ability to disrupt B cell pathways, thus possibly stimulating a T cell response that leads to the creation of a pro-inflammatory environment ^67^. In a clinical trial in patients with MS, atacicept was able to reduce the concentration of immunoglobulins and the number of circulating mature B cells correlating with an increment in relapses without changes in the CNS lesion load by MRI ^68^. Upon discontinuation of atacicept and a 60-week follow-up, laboratory findings and activity of disease normalized ^68^. In fact, atacicept and infliximab (a TNF blocking drug) have the ability to induce increment of memory B cells in the blood, enhance their ability to enter the CNS, and increase disease relapse rate and lesion load by MRI ^69^. Also, Badr *et al.* have reported evidence of synergy between BAFF and CXCL13 which could have important implications for homeostasis of B cells ^70^. Altogether these findings have led to conclude that the immune response in MS is unpredictable and complex and that additional studies most be conducted with significant focus on patient safety ^67, 68^.

## Would a complementary intrathecal therapy for deactivation of the mTLO be necessary to arrest disease progression?

A self-sustained intrathecal inflammation fostered by CSF chemokines involved in the traffic and survival of inflammatory cells occurs early in disease and is orchestrated by mTLO ^3^. Studies have shown that lineage of B cells can travel through peripheral blood, cervical lymphoid nodes, and the intrathecal compartment where they can be exposed to SMH in the mTLO and return to peripheral blood ^17^. As mentioned above, Piccio *et al*. found that CSF CXCL13 and CCL19 were decreased at week 24 after IV rituximab ^32^. However, Topping *et al*. found that therapy with intrathecal rituximab in patients with RRMS and SPMS resulted in no variation of CXCL13 levels in serum and CSF during the period of evaluation ^71^. Bonnan has hypothesized that, in order to prevent an unwanted generalized immune suppression resulting from systemic targeting of resident TLO, intrathecal immune reset should be attempted with a combination of monoclonal antibodies targeting each cell sub-type and aimed at eliminating simultaneously B cells, T cells, PC and FDC, via the intrathecal route. Excepting rituximab, candidate drugs still require preclinical studies for validation ^3^. Komori *et al*. reported that in patients with progressive MS who received therapy with intrathecal rituximab the depletion of B cells in the CSF compartment was transient and incomplete and could be facilitated by complement dependent cytotoxicity and to a lesser degree by antibody dependent celular cytotoxicity ^72^.

## Conclusion

An early neutralization of CXCL13 could interfere with the organization and function of the mTLO thus modifying and reducing inflammation in the CNS of patients with MS. Studies in animal models where CXCL13 has been neutralized, or is not expressed (such as the CXCL13-/- mice), confirm its crucial role maintaining, rather than initiating, inflammation and its manipulation could lead to modification of disease in these models ^39^. However, any therapeutic strategy unable to neutralize LLPCs or antibody secreting cells will not be successful in an attempt to impede the chronic progression of disease ^73^. Neutralization of the CXCL13 should be carefully sought as complementary therapy to the DMT in MS.

## Data availability

No data is associated with this article.
