# Supplementary material for: Role of CXCL13 in the formation of the meningeal tertiary lymphoid organ in multiple sclerosis
Source: F1000Res. 2018 Oct 4;7:514. Originally published 2018 Apr 27. [Version 3] doi: 10.12688/f1000research.14556.3 (PMC6171727; doi:10.12688/f1000research.14556.3)
Supplement: Peer review history

# Peer review: Referee response for version 3

**DOI:** 10.5256/f1000research.17962.r39063

**Published:** 2018-10-04

**Authors:** Hans Lassmann

**Affiliations:** 1 Center for Brain Research, Medical University of Vienna, Vienna, Austria

I have no new comments.

I have read this submission. I believe that I have an appropriate level of expertise to confirm that it is of an acceptable scientific standard.

# Peer review: Referee response for version 2

**DOI:** 10.5256/f1000research.17713.r38354

**Published:** 2018-09-24

**Authors:** Anneli Peters

**Affiliations:** 1 Institute of Clinical Neuroimmunology, Hospital and Biomedical Center of the Ludwig-Maximilians University, Martinsried-Planegg, Germany

The authors have addressed the concerns raised by the reviewers in the revised version.

 In the new version there is one misleading statement in the paragraph about TLOs becoming operation centers (pg 5): "In ..EAE they found the following: 1) Expression of CXCL13 by Th17 cells in the CNS;". This statement is not correct - in this reference CXCL13 expression was detected in the CNS of Th17 recipient mice, but the source of CXCL13 are (most likely) not Th17 cells themselves but other cell types like stromal cells. This should be corrected.

I have read this submission. I believe that I have an appropriate level of expertise to confirm that it is of an acceptable scientific standard.

# Peer review: Referee response for version 2

**DOI:** 10.5256/f1000research.17713.r38355

**Published:** 2018-09-17

**Authors:** Hans Lassmann

**Affiliations:** 1 Center for Brain Research, Medical University of Vienna, Vienna, Austria

The authors have revised the manuscript and have addressed all concerns raised by the reviewers.

I have read this submission. I believe that I have an appropriate level of expertise to confirm that it is of an acceptable scientific standard.

# Peer review: Referee response for version 1

**DOI:** 10.5256/f1000research.15843.r35347

**Published:** 2018-07-16

**Authors:** Anneli Peters

**Affiliations:** 1 Institute of Clinical Neuroimmunology, Hospital and Biomedical Center of the Ludwig-Maximilians University, Martinsried-Planegg, Germany

In this review article the authors describe the role of the chemokine CXCL13 in the formation of meningeal TLOs in MS and suggest it as a therapeutic target. The article is well written and the first half of the article provides a very detailed overview of the components and requirements for formation of secondary lymphoid organs. The authors then switch to tertiary lymphoid organs in the CNS assuming that all components and mechanisms of formation are identical to SLOs. While this may be the case for some of the most developed TLOs in some autoimmune diseases like Myasthenia gravis, it is not so clear which cell types and molecular players are required for formation of meningeal TLOs. In fact, to my knowledge it has not even been formerly proven that CXCL13 is required for formation of meningeal TLOs. Even though it is quite likely considering detection of CXCL13 in mTLOs and elevated CXCL13 levels in the CSF of MS patients, definitive proof even in the animal model is missing as also pointed out by reviewer 1, because a) CXCL13-deficient mice already have a defect in mounting proper immune responses in SLOs and b) active EAE induced by MOG-peptide/CFA immunization does not prominently feature mTLOs. The mouse models that do feature mTLOs such as the spontaneous 2D2xTh mouse have not been studied in the context of CXCL13 deficiency.

 Furthermore, it would be very useful to discuss in this review cellular sources of CXCL13 in the CNS, as they may not be identical to SLOs. Thus, microglia (Ref 37) and meningeal stromal cells (Pikor et al., Immunity, 2015 ^1^) have been suggested as sources for CXCL13 and should be discussed.

 Another important point is that the authors state that the "mTLO maintain differentiation and maturation of antigen-specific lymphocytes which perpetuate inflammation and disease progression". This is not a fact but a hypothesis and should be stated as such. While it is clearly an attractive hypothesis there is no proof neither in mouse models, nor in MS. We agree with the authors that in MS occurence of TLOs has been *associated *with more severe disease course and cortical lesions, however, causality has not been demonstrated and even evidence for maturation of antigen-specific lymphocytes in mTLOs is very limited so far. Therefore, we believe that it is not justifiable to interfere with mTLO formation in MS patients, as long as their biological function and consequences are not much better understood.

 As a side note, some sentences are a bit unclear, for example in the introduction "Inflammation is the appropriate immune response to...autoimmunity..." (pg 2) and "Tfh cells are characterized by expression of CXCR5 and ICOS, which is a subtype of Tfh cells" (pg 3).

 Overall, the review has a very interesting and important topic, however, in my opinion as detailed above in several paragraphs the wording should be a bit more careful and precise in order not to be misleading.

I have read this submission. I believe that I have an appropriate level of expertise to confirm that it is of an acceptable scientific standard, however I have significant reservations, as outlined above.

## References

[1] PikorNBAstaritaJLSummers-DelucaLGaliciaGQuJWardLAArmstrongSDominguezCXMalhotraDHeidenBKayRCastanovVTouilHBoonLO'ConnorPBar-OrAPratARamagliaVLudwinSTurleySJGommermanJL: Integration of Th17- and Lymphotoxin-Derived Signals Initiates Meningeal-Resident Stromal Cell Remodeling to Propagate Neuroinflammation. *Immunity*.2015;43(6) : 10.1016/j.immuni.2015.11.010 1160-73 10.1016/j.immuni.2015.11.010 26682987

# Response

**Published:** 2018-09-10

**Authors:** Carlos Mora

**Affiliations:** Georgetown University Hospital, Washington DC, USA

**Author response to Reviewer 2.**

We also thank Dr. Peters for her important observations to the content of the first version of the manuscript. We concur with the fact that the current knowledge on the formation of the secondary lymphoid organs (SLO) should not be unquestionably extrapolated and applied to the understanding of the genesis of the tertiary lymph nodes (TLO) especially in the context of neuroinflammation. We also understand that some of the existing concepts on this topic should still remain at a hypothetical, instead of conclusive, level of consideration. Yet, thanks to this reviewer comments and encouragement, especially in relation to discussion of possible cellular sources of CXCL13 in the CNS and to the search for further literature supporting a role for CXCL13 in the EAE animal models, we were able to expand in depth the content of the manuscript and bring more interesting material to the discussion giving further support to the role of this chemokine in the pathogenesis of MS. Certainly, patient safety is a demanding priority and any application of the knowledge acquired from *in vitro* or EAE animal model studies to the treatment of patients with MS should be considered with extreme caution.

# Peer review: Referee response for version 1

**DOI:** 10.5256/f1000research.15843.r33520

**Published:** 2018-04-30

**Authors:** Hans Lassmann

**Affiliations:** 1 Center for Brain Research, Medical University of Vienna, Vienna, Austria

In the review article the authors highlight the potential importance of tertiary lymph follicle like structures in the meninges of MS patients as driving forces for tissue damage and in particular cortical demyelination. They provide a very good summary of immunological mechanisms, which are involved in the organization and function of secondary lymph follicles in the peripheral lymphatic tissue and in particular highlight the importance of the interaction of CXCL13 with other cytokines and chemokines in these processes. They then describe in detail the evidence for the presence of structures with features of tertiary lymph follicles in the meninges of MS patients and their association with disease severity and cortical pathology. Finally they also review in detail the observations that CXCL13 is present in the CSF and may serve as a biomarker associated with poor prognosis of the disease. Based on the experimental observation that CXCL13 blockade or genetic ablation ameliorates EAE the authors propose that therapeutic blockade of CXCL13 in the CNS compartment of MS patients may be beneficial.

                 There is now good cumulative evidence that such follicle like inflammatory aggregates in the meninges are an important substrate of disease pathology in MS and that B-lymphocytes play an important, but so far not fully understood pathogenetic role in the disease. It is also clear that CXCL13 is an important chemokine, involved in B-cell recruitment into the central nervous system. However, it may be premature at the present time to propose intrathecal CXCR13 blockade as a therapy for MS patients. The EAE studies are only of limited value. It is not a surprise to ameliorate EAE with a therapy, which has major effects on the organization and function of peripheral lymphatic tissue. Although some EAE models show lymphocytic aggregates in the meninges, which share some features with those in MS, this is not the case in the majority of the models. Furthermore, in the respective mouse EAE models with lymph follicle like aggregates in the meninges there is no cortical demyelination. Thus lesion pathogenesis apparently is quite different between these models and MS. To what extent an intrathecal blockade of CXCL13 has an effect on CNS inflammation and what kind of effect will be achieved, is currently unknown. Whether this may induce dangerous side effects is also unclear. The suggestion to combine such a treatment with simultaneous intrathecal elimination of B-cells, T-cells and other immune cells is also far away from realization. Elimination with currently used antibodies requires complement of antibody dependent cellular cytotoxicity, and whether this is safe to induce within the CNS compartment in patients is also rather uncertain.

 Thus this review addresses a topic, which is interesting in a disease such as multiple sclerosis, but the suggestions for therapeutic translation are currently premature and potentially dangerous.

I have read this submission. I believe that I have an appropriate level of expertise to confirm that it is of an acceptable scientific standard, however I have significant reservations, as outlined above.

# Response

**Published:** 2018-05-30

**Authors:** Carlos Mora

**Affiliations:** Georgetown University Hospital, Washington DC, USA

We thank Dr. Lassmann (Referee 1) for the valuable comments addressed upon the review of version No. 1 of our article. We agree on the inconclusive current state of knowledge on the possible effect of intrathecal blockade of CXCL13 during CNS inflammation. On the concept of efficacy of monoclonal antibodies as modulators of the immune response in the cerebrospinal fluid (CSF), special mention deserves the work reported by Komori et al. on the insufficient inhibition of activity of disease, upon the administration of intrathecal rituximab, in chronic progressive multiple sclerosis (MS). These investigators found out that the efficacy of a monoclonal antibody in CSF will not be substantial as long as the blood brain barrier remains closed. Following rituximab therapy, depletion of B-cells in the CSF was facilitated by complement dependent cytotoxicity (CDC) and, to a lesser degree, by antibody dependent cellular cytotoxicity. Although a decrement in the concentration of complement would reduce the efficacy of CDC, the addition of complement in the CSF could lead to adverse effects in the CNS tissue [reference: Komori M, Lin YC, Cortese I, Blake A, Ohayon J, Cherup J, et al. Insufficient disease inhibition by intrathecal rituximab in progressive multiple sclerosis. Ann Clin Transl Neurol 2016;3(3):166-179 doi: 10.1002/acn3.293]. In relation to the concept of ‘therapeutic translation’ mentioned in the review, specifically on the effect of an eventual combination of intrathecal blockade of CXCL13 with simultaneous intrathecal elimination of B-cells, T-cells and other immune cells, we do agree this therapeutic approach would be premature and could be potentially harmful for the recipients of such combined therapies clarifying that the hypothesis formulated by Bonnan [ref. 3 in the article] does not make any reference to the intrathecal blockade of CXCL13 in MS. We look forward to hearing further comments from reviewers prior to publication of version No. 2 of the article.

# Response

**Published:** 2018-06-05

**Authors:** Hans Lassmann

**Affiliations:** Department Neuroimmunology, Medical University of Vienna, Austria

I aree with the comment of the authors.
